# Healthcare Expenditure and Productivity Cost Savings from Reductions in Cardiovascular Disease and Type 2 Diabetes Associated with Increased Intake of Cereal Fibre among Australian Adults: A Cost of Illness Analysis

**DOI:** 10.3390/nu10010034

**Published:** 2018-01-02

**Authors:** Flavia Fayet-Moore, Alice George, Tim Cassettari, Lev Yulin, Kate Tuck, Lynne Pezzullo

**Affiliations:** 1 Nutrition Research Australia, Level 13 167 Macquarie Street, Sydney, NSW 2000, Australia; tim@nraus.com (T.C.); kate@nraus.com (K.T.); 2 Deloitte Access Economics, 8 Brindabella Circuit, Brindabella Business Park, Canberra Airport, Canberra, ACT 2609, Australia; algeorge@deloitte.com.au (A.G.); lyulin@deloitte.com.au (L.Y.); lpezzullo@deloitte.com.au (L.P.)

**Keywords:** dietary fibre, cereal fibre, cardiovascular disease, type 2 diabetes, cost-of-illness analysis, public health, nutrition economics

## Abstract

An ageing population and growing prevalence of chronic diseases including cardiovascular disease (CVD) and type 2 diabetes (T2D) are putting increased pressure on healthcare expenditure in Australia. A cost of illness analysis was conducted to assess the potential savings in healthcare expenditure and productivity costs associated with lower prevalence of CVD and T2D resulting from increased intake of cereal fibre. Modelling was undertaken for three levels of increased dietary fibre intake using cereal fibre: a 10% increase in total dietary fibre; an increase to the Adequate Intake; and an increase to the Suggested Dietary Target. Total healthcare expenditure and productivity cost savings associated with reduced CVD and T2D were calculated by gender, socioeconomic status, baseline dietary fibre intake, and population uptake. Total combined annual healthcare expenditure and productivity cost savings of AUD$17.8 million–$1.6 billion for CVD and AUD$18.2 million–$1.7 billion for T2D were calculated. Total savings were generally larger among adults of lower socioeconomic status and those with lower dietary fibre intakes. Given the substantial healthcare expenditure and productivity cost savings that could be realised through increases in cereal fibre, there is cause for the development of interventions and policies that encourage an increase in cereal fibre intake in Australia.

## 1. Introduction

Chronic diseases, such as cardiovascular disease (CVD) and type 2 diabetes (T2D), have been regarded as Australia’s biggest health challenge and are the leading cause of illness, disability and mortality [1]. As a result of heath expenditures and lost productivity from increased morbidity and mortality, these diseases bring a significant economic burden to individuals and government [1]. Strategies to lower the prevalence of these diseases and their associated costs are increasingly important. Cost-of-illness studies are essential in developing the rhetoric for these strategies. In Australia, total healthcare expenditure has more than doubled since 2003 [2] and it is predicted to more than triple in the coming years, from AUD$129.7 billion in 2011–12 to AUD$416.3 billion in 2031–32 [3]. In the most recent Australian Health Survey, 6.7% of all adults had CVD and 5.7% had T2D [4]. Healthcare expenditure on CVD accounted for 12% of all allocated healthcare expenditure, more than other any disease group [5], and the proportion of the total expenditure attributable to both CVD and T2D is predicted to rise [3].

Given the substantial burden of CVD and T2D, interventions to reduce their impact on the Australian population are needed. Diet is considered one of the most important priorities in reducing the risk of CVD and T2D [6], and it has been confirmed in randomised controlled trials to reduce the incidence of both CVD events [7] and T2D [8] in at risk individuals. There is consistent evidence from systematic reviews and meta-analysis of cohort studies that greater dietary fibre intake is associated with a reduced risk of both CVD [9] and T2D [10,11,12]. Hence, dietary modification has an important role in reducing the economic costs associated with these chronic diseases. The dietary fibre from grain (cereal) foods is particularly important for chronic disease risk reduction. Comparisons of different fibre sources in epidemiological studies show that cereal fibre is associated with a greater risk reduction for T2D [10,11], and also possibly for coronary heart disease [9,13], than fibre from fruit and vegetables. A recent cost-of-illness analysis from Canada reported that the reduced prevalence of CVD and T2D expected from increasing cereal fibre intake would bring healthcare expenditure and productivity cost savings, up to CAD$720 million for T2D and CAD$1.3 billion for CVD [14].

Grain (cereal) foods are the leading food group contributor to total dietary fibre in the Australian diet [15], yet most Australians are not meeting the Australian Dietary Guidelines daily recommended serves for this food group [16]. It was reported 66.6% of adult males and 75.6% of adult females did not meet the recommended serves in 2011–12 [16]. A subsequent report also found a 29% decline in the consumption of grain (cereal) foods between 2011 and 2014 [17]. Furthermore, 70.7% of adult males and 77.0% of adult females did not meet the recommended daily serves for fruit, and 96.5% of adult males and 94.8% of adult females did not meet the recommended serves for vegetables and legumes/beans [16], which are also food sources of dietary fibre. The Suggested Target for dietary fibre to reduce chronic disease risk is 38 g for men and 28 g for women [18]. While there are no reports on the percentage of Australians who are falling below these suggested dietary fibre targets, the 2011–12 National Nutrition and Physical Activity Survey reported a mean dietary fibre intake per day of 24.8 g for men and 21.1 g for women [15]. It is likely that many Australians have dietary fibre intakes well below recommended levels to reduce chronic disease risk.

The potential reduction in healthcare expenditure and productivity costs from CVD and T2D as a result of increasing dietary fibre intake to the suggested dietary targets to reduce chronic disease risk has not been quantified in Australia. It is necessary to investigate how the potential cost savings may differ according to dietary fibre intake and socioeconomic status (SES) as lower SES groups in Australia generally have a higher prevalence of these chronic diseases [19], and also have poorer diets [20]. Determining the ‘cost-of-illness’ associated with dietary changes is not only important for healthcare providers, it is also crucial for policy makers looking to develop effective, evidenced-based strategies to reduce growing healthcare expenditure.

This study aimed to determine the potential economic savings, in terms of healthcare expenditure and productivity costs associated with reduced rates of CVD and T2D expected from increasing intakes of cereal fibre among Australian adults. In addition, we aimed to determine how the economic savings would vary by gender, SES and varying levels of dietary fibre intake.

## 2. Materials and Methods

A cost-of-illness analysis was conducted to determine the potential savings in healthcare expenditure and productivity costs associated with a lower prevalence of cardiovascular disease (CVD) and type 2 diabetes (T2D) resulting from increased intake of cereal fibre. The analysis examined savings by gender, quartiles of dietary fibre intake, quintiles of socioeconomic status (SES), as well as various levels of population uptake. The analysis involved three steps: (1) determining the total dietary fibre intake and prevalence of CVD and T2D in Australia; (2) calculating the potential reduction in CVD and T2D prevalence under the identified scenarios; and (3) calculating the healthcare expenditure and productivity costs associated with CVD and T2D. The total expected savings in healthcare expenditure and productivity costs were then modelled and calculated under each scenario. All input parameters calculated for the analysis in steps 1 to 3 were part of the methodology of this study and are reported in Table 1. Ethics approval was not required for this study as it involved the use of data that was already published or previously collected under the Census and Statistics Act 1905.

### 2.1. Step 1: Determining the Total Dietary Fibre Intake and Prevalence of CVD (Cardiovascular Disease) and T2D (Type 2 Diabetes) in Australia

#### 2.1.1. Dietary Fibre Intake

The average daily intake of dietary fibre in 2011–12 was 24.8 g per day for men and 21.1 g per day for women [15]. To provide an assessment of how dietary fibre intake varies among the Australian population, we used data from the nationally representative 2011–12 National Nutrition and Physical Activity Survey (NNPAS) to calculate dietary fibre intake by population quartiles and socio-economic status by population quintiles for Australian adult males and females. The NNPAS was undertaken by the Australian Bureau of Statistics (ABS) as part of the 2011–13 Australian Health Survey [19]. Trained ABS interviewers interviewed survey participants, and all foods and beverages consumed in the 24 h prior to the interview day were recorded. A second interview was conducted by telephone on approximately two-thirds of participants. To maximise sample size for this study, only the first day of the 24-h dietary recall was used, for 9341 adults aged 19 years and over. Data were weighted to represent the Australian population with weightings provided by the ABS.

Mean dietary fibre intake in grams was calculated for each respondent, and quartiles of fibre intake were determined. The lowest quartile of dietary fibre intake (bottom 25%) consumed, on average, 11.1 g for men and 9.5 g for women per day, while the highest quartile (top 25%) consumed, on average, 41.7 g for men and 36.8 g for women per day (Table 1).

SES was defined based on quintiles of the Socio-Economic Indexes for Areas (SEIFA) [25]. SEIFA is a product developed by the ABS based on Census data that includes several factors such as education, employment, and income that ranks areas in Australia into quintiles according to relative socio-economic advantage or disadvantage. The lowest quintile corresponds to the most disadvantaged, and the highest quintile to the least disadvantaged. The lowest SES quintile consumed, on average, 21.7 g of dietary fibre per day, compared to 24.2 g per day for those in the highest SES quintile (Table 1).

Sensitivity analysis of the estimates of average daily intake of dietary fibre was undertaken to investigate the potential for under reporting, a known limitation of self-reported survey data collection. Survey participants with an energy intake to basal metabolic rate ratio of <0.9 were classified as ‘underreporters’ based on the Goldberg cut-off [26] which is the lower 95% confidence limit for a single day of data for a single individual. The mean fibre intake among all survey participants was 22.9 g; when underreporters were excluded, the mean fibre intake was 24.7 g. To enhance the overall representativeness of the sample, all participants were included in the analysis, with the understanding that mean fibre intakes may have been underestimated in a proportion of the respondents. At a population level, this is not expected to have a substantial impact on the overall findings.

#### 2.1.2. Prevalence of CVD and T2D

Disease prevalence for CVD and T2D in 2015–16 were derived from the 2014–15 Australian Health Survey conducted by the ABS [4]. The prevalence of CVD included total heart, stroke and vascular conditions such as ischaemic heart disease and stroke, but excluded some other diseases of the circulatory system such as hypertension, haemorrhoids and varicose veins. A population growth rate of 1.75% was derived using the ABS Australian Demographic Statistics [27] and applied to determine the expected prevalence in 2015–16, which represented the growth in Australia’s population from the end of June quarter 2015 to the end of June quarter 2016. The total prevalence for CVD and T2D was estimated to be over 1 million persons for each disease in 2015–16 (Table 1). Disease prevalence was also obtained by SES, which was derived from the 2014–15 Australian Health Survey [4]. Disease prevalence was generally higher for the lower SES groups (Table 1).

### 2.2. Step 2: Calculating the Potential Reduction in CVD and T2D Prevalence under the Identified Scenarios

#### 2.2.1. Scenarios for Increased Intake of Cereal Fibre

Three separate scenarios were modelled to evaluate the effects of increased cereal fibre intake (Table 1). As this analysis used risk reductions associated with cereal fibre intake, all scenarios assumed that increases in dietary fibre intake were attributed solely to increased cereal fibre intake. The first scenario was a 10% increased (10% higher) above the average dietary fibre intake, which provides an example of a more conservative increase in cereal fibre consumption. The second scenario was an increase in fibre intake to Adequate Intake (adequate). The Adequate Intake for dietary fibre is set by the National Health and Medical Research Council (NHRMC) and is 30 g per day for men and 25 g per day for women [18]. The “adequate” scenario represents increases of 21.0% and 18.5% above the average daily fibre intake for men and women, respectively. The third scenario was an increase in cereal fibre intake to the Suggested Dietary Target for dietary fibre for reducing the risk of chronic disease (target), also set by the NHMRC, and is 38 g per day for men and 28 g per day for women [18]. The “target” scenario represents increases of 53.3% and 32.7% above the average daily fibre intake for men and women, respectively.

We modelled increases in fibre using cereal fibre for a number of reasons. First, when the source of dietary fibre is examined, the risk reduction for type 2 diabetes is much greater for grain fibre than fruit or vegetable fibre: per 10 g/day increase in intake, the risk reduction was 0.75 (95% CI 0.65, 0.86) for cereal fibre, 0.95 (95% CI 0.87, 1.03) for fruit fibre and 0.93 (95% CI 0.82, 1.05) for vegetable fibre [10]. Thus, acknowledging the source of fibre is important for achieving an accurate estimation in the potential disease reduction and thus economic savings. Second, the majority of Australians consume grain foods and these are the leading source of dietary fibre in the Australian diet [15]. In contrast, the vast majority (≥95%) of Australian adults did not meet the Australian Dietary Guideline targets for daily serves of vegetables or legumes/beans [16], highlighting that some fibre sources may be difficult to increase. Third, while the encouragement of fruits and vegetables has previously been the subject of national health campaigns [28] and are likely well known to be health promoting, there is significant confusion surrounding the health benefits of grain foods [17]. Targeted interventions encouraging high-fibre grain food consumption may therefore be more susceptible to change, particularly as the majority of current grain food intake comes from refined or lower fibre grain foods [16] and so increasing cereal fibre intake can be realised through simple dietary swaps.

#### 2.2.2. Calculating the Potential Reduction in CVD and T2D Prevalence

To calculate the potential reduction in CVD and T2D prevalence, we searched for recent dose-response meta-analyses of prospective cohort studies that reported the risk reduction for CVD and T2D associated with cereal fibre intake. For T2D, we applied estimates of disease risk reduction from InterAct Consortium 2015 [10]. The research estimated a 25% reduction in the risk of T2D for a 10-g average daily increase in cereal fibre (RR = 0.75, 95% CI: 0.65–0.86). This is equivalent to a 2.5% risk reduction per 1 g (Table 1). For CVD, we applied estimates of disease risk reduction from Threapleton et al. 2013 [9]. Cereal fibre reduced the risk of CVD by 8% for a 7-g average daily increase (RR = 0.92, 95% CI: 0.84 to 1.00), which is equivalent to a 1.1% risk reduction per 1 g (or 11% per 10 g) (Table 1).

Using these data, we calculated the number of cases averted by assuming that the Australian prevalence of CVD and T2D would be lower by 1.1% and 2.5%, respectively, for each one gram increase in cereal fibre intake, as reported in Supplementary Table S1. The scenarios were modelled to reflect that cereal fibre intake had been increased historically so that expected reductions in CVD and T2D prevalence were fully realised in 2015–16. For the purpose of this modelling, we assumed that the relative risk reduction of CVD and T2D per gram cereal fibre was linear. While there was evidence of non-linearity in the association between cereal fibre intake and risk reduction of T2D, given that this relationship showed a steeper reduction in risk at higher levels of intake, assuming a linear association is considered to be a conservative estimate, particularly at higher levels of intake.

The number of cases averted for each scenario was calculated by multiplying the difference in fibre intake by the risk reduction (1.1% for CVD and 2.5% for T2D per gram) and by the estimated prevalence of CVD and T2D. The percentage reduction in disease prevalence was calculated by dividing the estimated number of cases averted by the total prevalence of CVD and T2D.

### 2.3. Step 3: Calculating the Healthcare Expenditure and Productivity Costs Associated with CVD and T2D

#### 2.3.1. Population Uptake Analysis

Four population uptake scenarios were modelled to assess the potential healthcare expenditure and productivity cost savings by varying levels of increased cereal fibre intake across the Australian population. These were universal, optimistic, pessimistic and very pessimistic uptakes, and were based on scenarios from previous research [14]. Universal uptake assumed a 100% uptake rate that provides an indication of the maximal potential savings that could be realised. Optimistic uptake assumed a 50% uptake by the Australian population as a longer-term, pragmatic estimate. The pessimistic and very pessimistic success rates were set at 15% and 5% respectively, and represent a less positive but more realistic short-term estimate. The resulting impact on healthcare expenditure and productivity cost savings was modelled for the 2015–16 financial year and included sub-group analysis by gender and SES.

#### 2.3.2. Healthcare Expenditure

We estimated reductions in healthcare expenditure by calculating the average expenditure per prevalent case for CVD and T2D in 2015–16. In 2008–09, CVD and T2D accounted for $7.6 billion [5] and $0.9 billion [21] in healthcare expenditure respectively. We accounted for rates of inflation by multiplying this healthcare expenditure by the growth in the health price index, which is a measure of the change in average health prices from year to year at the national level. Since the health price index was only available up to 2013–14 [2], we used the average historical growth rate per year over the previous 10 years (2004–05 to 2013–14) calculated as 2.6%, and applied this average growth rate for the years 2014–15 and 2015–16. The estimated healthcare expenditure from these calculations is presented in Table 1. The same calculations were also applied for the sub-categories of healthcare expenditure (i.e., hospital-admitted patient services, out-of-hospital medical expenses and prescription pharmaceuticals). The healthcare expenditure per case was then estimated by dividing the total healthcare expenditure by the number of prevalent cases (Supplementary Table S2). The expenditure for both CVD and T2D was based only on recurrent expenditure related to goods or services that are consumed within a short time frame, and excludes fixed costs that are common to many diseases including certain assets and administration.

#### 2.3.3. Indirect (Productivity) Costs

Indirect costs of lost productivity to society arise from mortality and morbidity of CVD and T2D. These include: absenteeism, where workers are absent from work due to CVD or T2D; presenteeism, representing lost productivity due to people attending work while they are experiencing poor health; premature death, resulting in lost productivity and lifetime earnings of workers who die prematurely because of CVD or T2D; and reduced labour force participation, which includes lost productivity associated with lower levels of employment due to people having CVD and T2D.

Indirect costs were calculated using data on lost productivity and premature death costs per prevalent case of CVD [22] and diabetes (types 1 and 2) [23] and are presented in Table 1. Due to limitations in the availability of data by type of diabetes, the productivity costs relate to both type 1 and type 2 diabetes. It is noted T2D comprises 86.2% of total diabetes prevalence, and hence the productivity costs per case of diabetes was used to approximate the costs for T2D specifically [4]. A human capital approach was used to estimate productivity costs from people with CVD or diabetes compared to the general population [29]. Employment activity by people with CVD and diabetes were derived using available data from the National Health Survey [30] and the Disability, Ageing and Carers Survey [31], respectively. The productivity costs from people who die prematurely was estimated based on the assumption that if they had lived, the person would have earned an average annual income up until their retirement at the same rate as the age-standardised general population. This represents the discounted lost lifetime earnings. The costs of presenteeism for CVD were calculated based on previous research on presenteeism for stroke, due to limitations in available data for CVD as a whole [24]. Stroke contributed to approximately 14.5% of the total prevalence of CVD [4]. All productivity costs were inflated to 2015–16 figures using growth in the ABS Wage Price Index over the previous years [32]. This was a growth rate of 2.3% for 2014–15 and 2.1% for 2015–16.

Productivity costs per case were calculated by dividing the total estimated productivity costs for CVD and T2D by the prevalence of each disease (Supplementary Table S2). It is noted that the prevalence estimate for CVD in the 2005 Access Economics report [22] was 3.2 million, and that differences in the our prevalence estimates arise from different categorisation of CVD. The present analysis uses the definition of CVD in terms of ‘Total heart, stroke and vascular diseases [4], while the 2005 Access Economics report used the definition ‘Total diseases of the circulatory system’, which includes conditions such as hypertension, haemorrhoids and varicose veins. The prevalence of diabetes (types 1 and 2) was estimated at 1.1 million people in 2014. Total productivity cost savings from absenteeism, presenteeism, and reduced labour force participation were then calculated by applying the cost per case to the estimated reduction in the prevalence of CVD and T2D. Due to limitations in published data, a cost per prevalent case was not available separately by gender or SES.

#### 2.3.4. Costs of Premature Deaths from CVD and T2D

A search of the literature for dose-response meta-analyses of prospective cohort studies was conducted to quantify the total potential cost savings of premature death. For CVD, data were used from a dose-response meta-analysis conducted by Kim et al. 2016 [33]. They estimated a 9% reduction in the risk of CVD mortality from a 10 gram per day increase in total dietary fibre intake (RR = 0.91, 95% CI: 0.88 to 0.94), but did not report on cereal fibre specifically. Although the reduced risk of death from CVD associated with cereal fibre intake had been reported by Hajishafiee et al. 2016 for highest vs. lowest intake of cereal fibre (RR = 0.82, 95% CI: 0.78–0.86) [34], we applied data from Kim et al. 2016 as they reported on dose-response studies. The risk reduction reported by Kim et al. 2016 was then extrapolated to indicate a 0.9% reduced risk of CVD mortality per 1 gram increase in cereal fibre intake, and was applied against the number of deaths from CVD estimated in 2015–16. This was estimated from ABS Australian Demographic Statistics 2014 data [35] and the rate of growth in the Australian population. There were an estimated 22,012 men and 23,818 women (45,830 persons) who died from CVD in 2015–16. The costs of premature deaths were then divided by the estimated number of deaths from CVD to provide an indication of the potential cost per premature death. The cost per death was inflated to 2015–16 using growth in the ABS Wage Price Index [32] and was estimated to be $42,274.30 per death averted in 2015–16 (Supplementary Table S2).

There were no published data from systematic literature reviews or meta-analyses on the reduced risk of death from diabetes associated with increased dietary fibre intake. The cost of premature deaths averted from increased cereal fibre intake was therefore estimated using the relationship between prevalence and mortality of T2D, applied against the number of prevalent cases averted. The productivity cost per case of premature death from diabetes (both types 1 and 2) was used to represent the cost of premature death for T2D [23]. Again, premature death costs per prevalent case were inflated to 2015–16 figures using growth in the ABS Wage Price Index over the previous year [32]. Therefore, the productivity costs of premature death per prevalent case of T2D were estimated to be $255.13 per person (Supplementary Table S2). This was applied to the estimated number of prevalent cases averted, to derive potential productivity cost savings of premature death.

## 3. Results

### 3.1. The Total Expected Savings in Healthcare Expenditure and Productivity Costs under Each Scenario

#### 3.1.1. Healthcare Expenditure Savings

Table 2 presents the healthcare expenditure savings from increased cereal fibre intake for both cardiovascular disease (CVD) and type 2 diabetes (T2D) by gender, quintiles for socio-economic status (SES) and quartile of dietary fibre intake. Healthcare expenditure on CVD could be lowered by $221.5 million from a 10% higher intake, $467.3 million at the adequate intake, and up to $1.03 billion from the target intake level. For T2D, potential healthcare expenditure savings were estimated to be $60.7 million, $131.0 million and $285.9 million for 10% higher than the average intake, adequate intake and target intake, respectively. The potential healthcare expenditure savings were generally greater among the lower SES quintiles than the higher SES quintiles, which can be explained by the lower SES groups typically having a higher prevalence of CVD and T2D and a lower dietary fibre intake, However, this relationship is not observed uniformly across the groups because the prevalence of both CVD and T2D did not always decrease among the higher SES quintiles.

Potential healthcare expenditure savings from increased fibre intake from cereal fibre was calculated by category of healthcare expenditure for CVD and T2D (Supplementary Table S3). Hospital-admitted patient services contributed to the largest expenditure saving for both diseases, followed by prescription pharmaceuticals.

#### 3.1.2. Productivity Cost Savings

Table 3 summarises the potential indirect savings from increased productivity for CVD and T2D by gender, SES and quartiles of fibre intake arising from increased fibre intake from cereal fibre. Productivity cost savings for CVD were calculated to be $134.8 million for a 10% higher intake, $278.8 million for the adequate intake, and $609.5 million for the target intake. For T2D, the potential savings from increased productivity ranged from $302.6 million, $652.6 million and $1.4 billion for 10% higher intake, adequate intake and target intake, respectively. The higher productivity cost savings for T2D in comparison to CVD were partly a result of a greater risk reduction per gram of cereal fibre intake and a higher cost per case. Like healthcare expenditure savings, productivity cost savings were generally higher for lower SES groups for nearly all levels of increased cereal fibre intake for both CVD and T2D.

The potential productivity cost savings for CVD were estimated to be between $57.0 million for 10% higher and up to $590.2 million for target intake for all quartiles of dietary fibre intake. Productivity cost savings for T2D were estimated to be $175.0 million for 10% higher intake and up to $1.6 billion for the target intake. The highest quartile of dietary fibre intake was not considered to contribute to any productivity cost savings.

Potential productivity cost savings from increasing fibre intake using cereal fibre were also determined by category of productivity cost savings for CVD and T2D (Supplementary Table S3). Reduced presenteeism contributed to the largest expenditure saving for both diseases, followed by increased employment for T2D and premature deaths averted for CVD, for all levels of increased cereal fibre intake.

#### 3.1.3. Healthcare Expenditure, Productivity Cost and Combined Savings by Population Uptake

Table 4 shows the potential healthcare expenditure, productivity cost and combined savings that could be realised if varying shares of the Australian population increased their cereal fibre intake. Total savings of $1.6 billion for CVD and $1.7 billion for T2D, for both healthcare expenditure and productivity cost savings combined, could be realised under “universal” (100%) population uptake of the target intake level. Under the target intake model, healthcare expenditure savings for the “very pessimistic” (5%) population uptake were estimated to be $51.3 million for CVD and $14.3 million for T2D, and for the “universal” population uptake, $1.03 billion for CVD and $285.9 million for T2D. For productivity cost savings, a ‘universal’ population uptake to target intake would result in savings of $609.5 million for CVD and $1.4 billion for T2D.

The potential healthcare expenditure and productivity cost savings was also calculated based on a one-gram increase in cereal fibre intake, by the varying population uptake scenarios (Supplementary Table S4). If the entire Australian population increased their intake of cereal fibre by one gram per day, the total healthcare expenditure savings were estimated to be $96.5 million for CVD and $26.5 million for T2D. For productivity cost savings, up to $58.9 million for CVD and $132.3 million for T2D were estimated.

## 4. Discussion

There were substantial potential economic savings, both healthcare expenditure and productivity costs, from using cereal fibre intake to increase dietary fibre intake among Australian adults. The combined healthcare expenditure and productivity cost savings ranged between AUD$17.8 million–$1.6 billion for cardiovascular disease (CVD) and AUD$18.2 million–$1.7 billion for type 2 diabetes (T2D). The highest savings assumed a universal uptake of increased cereal fibre to the Suggested Dietary Target to reduce the risk of chronic disease. Up to $1.6 billion in savings for CVD and $1.7 billion for T2D could ultimately be realised annually if all adult Australians used cereal fibre to increase their total dietary fibre to the target intake level. Healthcare expenditure savings at the universal population uptake were $1 billion for CVD and over $285 million for T2D, which represents savings of approximately 0.6% and 0.2% of the total healthcare expenditure in Australia respectively [36]. The potential productivity cost savings at the universal uptake level were estimated to be approximately $600 million for CVD and $1.4 billion for T2D, which represents around 0.04% and 0.08% of the gross domestic product (GDP) [37]. Across different levels of cereal fibre intake, total potential savings were generally larger for groups of lower SES and those with lower total dietary fibre intake.

The finding that substantial economic savings are likely with large-scale dietary changes that reduce chronic disease risk is consistent with a growing body of literature globally. Modelling studies have demonstrated significant healthcare expenditure savings from a range of dietary changes, including but not limited to reduced nutrient intakes (such as sodium, sugars, saturated and trans fat), or increased fruit and vegetable or dairy consumption, in the United States (US) [38,39], New Zealand [40,41], England [42,43,44], Germany [45] and Canada [46,47,48]. In Australia, low dairy consumption has previously been estimated to contribute to a substantial component of total healthcare expenditure [49], and increased fruit or vegetable consumption has been modelled to bring about economic savings via a reduction in BMI (body mass index) [50] and a reduction in cardiovascular disease and some cancers [51].

While the association between increased cereal fibre intake and chronic disease risk reduction is well established, there is a lack of data describing healthcare expenditure and productivity cost savings from increasing cereal fibre intake. An economic analysis on the potential savings from reductions in CVD and T2D following increased intakes of cereal fibre was modelled in Canada [14]. They estimated total savings of CAD$718.8 million for CVD and CAD$1.3 billion for T2D with a universal (100%) population uptake of increased cereal fibre intake at the Institute of Medicine recommended adequate intakes of 38 g per day for men and 25 g per day for women. Similar to the Australian estimates, these savings were considerable relative to healthcare expenditure. It is difficult to directly compare these findings to those we estimated for Australia due to differences in healthcare systems (healthcare is purely publicly funded in Canada), CVD and T2D prevalence rates, population fibre intake which is influenced by dietary habits and the food supply, and differences in currency.

Our findings were modelled based on reduced cases of CVD and T2D, and excluded other health benefits of cereal fibre, which is likely to underestimate the true economic benefits of increased cereal fibre intake. Evidence from prospective studies shows cereal fibre specifically reduces the risk of colorectal cancer [52], diverticular disease [53], weight gain [54,55] and all-cause mortality [34]. In addition, modelling studies in the US [56] and Canada [57] have demonstrated substantial savings in medical costs from a reduction in constipation with increased dietary fibre consumption, for which some types of cereal fibre are considered particularly efficacious [58]. Furthermore, our findings are modelled based on a population who are free of CVD and T2D, and not to those who already have these diseases. Evidence from intervention studies report that increasing fibre intake, including cereal fibre, can directly improve biomarkers such as cholesterol [59,60] and blood glucose and Haemoglobin A1c [61], which can be expected to translate to additional healthcare expenditure and productivity cost savings for those currently with these diseases.

Other economic savings not modelled in our study include factors related to lost wellbeing. The total cost of lost wellbeing, including factors such as pain and suffering, potential weight gain and overall reduced quality of life, has previously been estimated to be around $31.8 billion for CVD and $5.3 billion for T2D [62]. This is substantially higher than the total financial costs of $1.6 billion for CVD and $1.7 billion for T2D we estimated in this study, which included both healthcare expenditures and productivity costs.

While our modelling assumes an increase in cereal fibre intake only, increasing cereal fibre intake is also likely to bring about unintended changes to other aspects of the diet, which may influence the estimated economic savings. Messages to increase cereal fibre intake without affecting total kilojoule or carbohydrate intake may need to focus on swapping lower fibre for higher grain foods, or choosing ‘fibre-dense’ grains that have a high fibre to carbohydrate ratio. A study from the US found that selecting grain products based on a high fibre to carbohydrate ratio leads to more healthful choices for whole grain products when compared to a number of other selection strategies [63]. Further economic savings are expected if dietary fibre is also increased from other fibre sources, such as fruit and vegetables, which is inversely associated with a reduced risk of CVD [9]. Strategies aimed at improving total diet quality in line with dietary recommendations to reduce the risk of chronic disease have a greater potential to bring about significant economic savings.

Higher economic savings were realised from higher rates of population uptake and a greater increase in cereal fibre intake. These findings support the need for public health interventions that deliver large increases in cereal fibre and reach a large audience. Potential public health interventions are numerous and may include mass media campaigns, improving food supply, subsidisation of foods rich in cereal fibre, working with retailers to provide nutrition information at point of sale, and providing nutrition education in workplaces, schools and communities [64]. Behavioural change from such campaigns is considered difficult to maintain in the long-term [65]. For instance, a meta-analysis of mass media health campaigns aimed at improving diet in the US reported that campaigns were effective at changing the behaviour of about 8% of the population [66]. This lies between the pessimistic (15%) and very pessimistic (5%) success rates modelled in our analysis. There is evidence that more substantial changes are possible with combined strategic efforts. For example, it has been described that a collaborative and strategic approach involving health authorities, non-governmental organisations and the food industry in Denmark was associated with the usual intake of whole grains almost doubling from 2000–04 to 2011–13 [67]. It is likely that a similar collaborative effort is required in Australia to realise the potential savings modelled.

Despite the established health benefits of cereal fibre, the evidence on the effectiveness of strategies designed to increase its consumption is limited. Nutrition education and the delivery of positive health messages have been shown to be effective at increasing dietary fibre intake at the individual level. The provision of whole grain foods and delivery of positive health messages for their consumption has been shown to increase individual dietary fibre intake [68]. A randomised controlled trial that consisted of individual and group education sessions found recommendations to increase fibre intake to ≥30 g per day increased intake by 7.1 g per day, compared to a smaller increase in the control group who received general dietary advice about healthy eating [69]. This level of increase falls between the adequate intake and target intake that we modelled.

It is important to consider the costs of interventions aimed at increasing cereal fibre intake to ensure that they are also cost-effective. An analysis of Australian interventions reported that population-wide approaches to intervention, including changing product labelling, engaging the food industry, and conducting community mass media campaigns, are most likely to be cost saving to the health sector [70]. Nutrition interventions in workplaces and supermarkets and the majority of individually targeted behaviour change interventions were considered least likely to be cost-effective. Direct costs to the consumer also need to be considered. For instance, it has been described that healthier diets generally cost more than unhealthy diets [71] and international evidence suggests that bread products marketed as being whole grain are more likely to cost more than other bread products [72]. These costs could be considered marginal given grains are generally associated with a relatively low per-calorie food cost overall [71] and the potential longer-term healthcare expenditure and productivity cost savings from increased intake of cereal fibre are substantial.

Education programs and health messages could be targeted at population groups where the greatest health and economic benefits were modelled, such as lower socio-economic groups or those with lower dietary fibre intake. In Australia, lower SES groups generally have a poorer diet quality [20] and those living in low-income households were least likely to purchase foods that were comparatively high in fibre, related in part to food-cost concern [73]. A systematic review of interventions to promote healthy eating found the efficacy of the intervention type varied depending on SES [74]. Interventions based on price were most effective in groups with lower SES, while individual targeted interventions such as dietary counselling appeared to widen inequalities. For those with low dietary fibre intakes, consumer misinformation has been identified as a major barrier to meeting fibre recommendations [75]. In Australia there is thought to be a lack of awareness and widespread misunderstanding of the health benefits of grain (cereal) foods, with a consumer survey reporting that people limit their intake of grain foods in an attempt to avoid bloating and to assist with weight loss [17]. A recent secondary analysis of the 2011–12 National Nutrition Survey in Australia reported that dietary fibre intake was directly related to the number of serves of grain (cereal) foods [76]. Together, these findings suggest there is a great opportunity for government and policy makers to consider targeted interventions aimed at those of low SES or with lower fibre intakes, to improve cost-effectiveness and maximise economic savings.

### 4.1. Future Research Recommendations

Further research is necessary to better understand the extent to which various interventions are likely to reach a large population, result in a sustained increase in cereal fibre intake, minimise the risk of unintended consequences, and achieve a cost-effective implementation.

### 4.2. Strengths and Limitations

This study has a number of strengths. Cereal fibre intake modelling was based not only on the average dietary fibre intake, but also by varying levels of intake and by socioeconomic status. These data provide key insights into which groups hold the largest potential for cost savings and may help determine the most cost-effective interventions. The potential cost savings were also estimated across increased levels of dietary fibre intake using cereal fibre (10% higher, adequate intake and target intake), with varying rates of population uptakes.

Economic modelling studies have a number of limitations that need to be considered. The relationship between CVD, T2D and economic costs was assumed to be absolute, and does not account for additional savings associated with the decrease in the severity of new cases of CVD or T2D, such as a reduction in the dose and frequency of medications or the costs associated with management by an allied health professional. Our modelling assumed that the potential cost savings have been achieved in the present day, but these changes can take years to be realised. We assumed that the total prevalence of CVD and T2D is known and that the rate of prevalence in proportion to the total population remains constant. In fact, many cases of T2D remain undiagnosed and rates of T2D are predicted to rise substantially over the next one to two decades [77], meaning total economic savings may possibly be larger than the current estimated results. The relationship between cereal fibre and risk reduction for CVD and T2D was assumed to be linear. The meta-analysis for cereal fibre and T2D risk reduction found evidence of non-linearity, with a more marked reduction in risk at higher levels of cereal fibre intake [10]. Economic savings for those in the highest quartile of dietary fibre intake were not modelled since their average intake was higher than the target intake, although it is likely that some economic savings are still possible with further increases in cereal fibre intake. Further, it is possible that the dietary fibre intakes data underestimated actual dietary fibre intake. As with other nutrition surveys, dietary fibre intake from the 2011–12 NNPAS may have been affected by underreporting [15], and the fact that food composition databases may not capture all types of dietary fibres, such as resistant starch [18]. If dietary fibre intakes were underestimated, potential cost savings can be expected to be marginally higher for the 10% intake scenarios and lower for the adequate and target intake scenarios. Mean fibre intakes were determined from a single day intake of the 2011–12 NNPAS, rather than calculating usual intake, although the group means for each are likely to be similar [78]. Finally, our study assumes the relationship between increased cereal fibre intake and risk reduction for CVD and T2D is causal.

## 5. Conclusions

Substantial economic savings could be realised if Australian adults use cereal fibre to increase their intake of dietary fibre. Combined potential healthcare expenditure and productivity cost savings of AUD$1.6 billion for cardiovascular disease and AUD$1.7 billion for type 2 diabetes were modelled for a universal (100%) population uptake of increasing dietary fibre to the target intake level for reducing chronic disease risk. The potential savings were generally greatest for groups of low SES and for those with low dietary fibre intake. Given the rising prevalence of chronic diseases and their associated healthcare expenditure in Australia, these findings make a compelling cause for cereal fibre to be a key component of future policies aimed at improving the health and dietary intake of Australians.

## Figures and Tables

**Table 1:** Cost-of-illness analysis input parameters.

Input Parameter	Persons	Males	Females	Source
Step 1: Levels of total daily fibre intake				
Mean (g per day)	-	24.8	21.1	ABS, 2014 [15]
Fibre intake quartile 1 (lowest) (g per day)	-	11.1	9.5	ABS, 2014 * [15]
Fibre intake quartile 2 (g per day)	-	18.9	16.4	
Fibre intake quartile 3 (g per day)	-	26.4	22.6	
Fibre intake quartile 4 (highest) (g per day)	-	41.7	36.8	
SES quintile 1 (lowest) (g per day)	21.7	23.7	19.6	ABS, 2014 * [15]
SES quintile 2 (g per day)	22.3	24.1	20.4	
SES quintile 3 (g per day)	22.9	24.7	21.1	
SES quintile 4 (g per day)	23.3	25.4	21.1	
SES quintile 5 (highest) (g per day)	24.2	25.9	22.8	
Step 1: Disease prevalence				
CVD prevalence (number of persons in 2015–16)	1,206,953	651,709	555,244	ABS Australian Health Survey, 2014–15 [4]
SES quintile 1 (lowest)	317,179	171,265	145,914	
SES quintile 2	264,786	142,974	121,812	
SES quintile 3	236,977	127,959	109,019	
SES quintile 4	190,831	103,042	87,790	
SES quintile 5 (highest)	197,179	106,469	90,710	
T2D prevalence (number of persons in 2015–16)	1,021,362	559,727	461,635	ABS Australian Health Survey, 2014–15 [4]
SES quintile 1 (lowest)	310,681	170,259	140,421	
SES quintile 2	196,813	107,858	88,956	
SES quintile 3	218,691	119,847	98,844	
SES quintile 4	171,060	93,744	77,315	
SES quintile 5 (highest)	124,117	68,019	56,098	
Step 2: Fibre scenarios and potential reduction in CVD and T2D prevalence				
10% higher intake (g per day)	-	27.3	23.2	-
Adequate fibre intake (g per day)	-	30.0	25.0	NHMRC & NZ MoH, 2006 [18]
Target fibre intake (g per day)	-	38.0	28.0	NHMRC & NZ MoH, 2006 [18]
CVD risk reduction per one-gram cereal fibre intake (%)	1.1	-	-	Threapleton et al., 2013 [9]
T2D risk reduction per one-gram cereal fibre intake (%)	2.5	-	-	InterAct Consortium, 2015 [10]
Step 3: Healthcare expenditure and productivity costs associated with CVD and T2D				
Total direct healthcare expenditure CVD (AUD $m)	8795.0	-	-	AIHW, 2014 [5]
Hospital-admitted patient services	5157.9	-	-	
Out-of-hospital medical expenses	1731.2	-	-	
Prescription pharmaceuticals	1905.9	-	-	
Total direct healthcare expenditure T2D (AUD$m)	1061.6	-	-	AIHW, 2013 [21]
Hospital-admitted patient services	629.1	-	-	
Out-of-hospital medical expenses	202.4	-	-	
Prescription pharmaceuticals	229.0	-	-	
Estimated productivity costs CVD (AUD$m)	6212.4	-	-	Access Economics, 2005 [22]
Reduced employment	3248.9	-	-	
Premature death	1937.4	-	-	
Absenteeism	141.9	-	-	
Presenteeism ^†^	884.2	-	-	
Estimated productivity costs T2D ^††^ (AUD$m)	5681.0	-	-	Deloitte Access Economics, 2014 [23]
Reduced employment	1443.7	-	-	
Premature death	279.9	-	-	
Absenteeism	324.3	-	-	
Presenteeism	3633.1	-	-	

Abbreviations: AUD, Australian Dollar; CVD, cardiovascular disease; m, million; SES, socioeconomic status; T2D, type 2 diabetes; ABS, Australian Bureau of Statistics; NHMRC, National Health and Medical Research Council; NZ MoH, New Zealand Ministry of Health; AIHW, Australian Institute of Health and Welfare. ‘10% higher’ is a 10% increase in current fibre intakes and is equivalent to an increase of between 2.1–2.5 g per day; ‘adequate intake’ is an increase in current dietary fibre intake to 30 g per day for males and 25 g per day for females and is equivalent to an increase of 3.9–5.2 g per day; and ‘target intake’ is an increase in current dietary intake to 38 g per day for males and 28 g per day for females and is equivalent to an increase of 6.9–13.2 g per day. All scenarios increase fibre intake using cereal fibre. ^†^ Based on presenteeism costs for stroke; Deloitte Access Economics, 2013 [24]. ^††^ Based on data for type I and II diabetes. * Levels of total daily fibre intake were determined by fibre intake quartiles and socio-economic status quintiles from an analysis of the 2011–12 National Nutrition and Physical Activity Survey. [] see manuscript reference list.

**Table 2:** Healthcare expenditure savings for cardiovascular disease and type 2 diabetes (AUD $m).

Scenario for Increased Intake of Fibre	Socio-Economic Status						Dietary Fibre Intake				
	Total	Quintile 1 (Lowest SES)	Quintile 2	Quintile 3	Quintile 4	Quintile 5 (Highest SES)	Total	Quartile 1 (Lowest)	Quartile 2	Quartile 3	Quartile 4 (Highest) ^†^
Cardiovascular Disease											
Persons											
10% higher	221.5	55.6	47.6	43.8	35.8	38.6	127.9	25.1	43.0	59.8	-
Adequate	467.3	148.9	112.4	88.6	65.7	51.8	737.9	421.4	242.3	74.2	-
Target	1026.5	295.8	235.1	198.4	154.1	143.2	1120.2	523.2	382.5	214.5	-
Males											
10% higher	132.8	33.6	28.5	26.2	21.6	22.8	76.4	15.0	25.6	35.8	-
Adequate	290.2	89.0	69.5	56.0	39.4	36.3	455.0	256.0	150.6	48.4	-
Target	721.7	202.4	164.2	140.7	107.6	106.8	780.0	364.3	258.9	156.7	-
Females											
10% higher	88.7	22.0	19.1	17.6	14.2	15.8	51.5	10.1	17.4	24.0	-
Adequate	177.1	59.9	42.9	32.5	26.3	15.5	282.9	165.4	91.7	25.8	-
Target	304.8	93.4	70.9	57.6	46.4	36.4	378.7	197.4	123.6	57.7	-
Type 2 Diabetes											
Persons											
10% higher	60.7	17.7	11.5	13.1	10.4	7.9	35.1	6.9	11.8	16.4	-
Adequate	131.0	47.4	27.2	26.6	19.1	10.6	202.6	115.7	66.5	20.4	-
Target	285.9	94.5	57.0	59.8	45.1	29.5	318.8	154.4	105.3	59.1	-
Males											
10% higher	37.0	10.9	7.0	8.0	6.4	4.7	21.3	4.2	7.1	10.0	-
Adequate	82.2	28.8	17.1	17.1	11.7	7.5	126.6	71.3	41.9	13.5	-
Target	202.8	65.5	40.3	42.9	31.9	22.2	217.1	101.4	72.1	43.6	-
Females											
10% higher	23.7	6.8	4.5	5.2	4.0	3.2	13.8	2.7	4.7	6.5	-
Adequate	48.8	18.6	10.1	9.5	7.5	3.1	76.0	44.4	24.6	6.9	-
Target	83.1	29.0	16.7	16.9	13.2	7.3	101.7	53.0	33.2	15.5	-

Abbreviations: SES, socioeconomic status. ‘10% higher’ is a 10% increase in current fibre intakes and is equivalent to an increase of between 2.1–2.5 g per day; ‘adequate intake’ is an increase in current dietary fibre intake to 30 g per day for males and 25 g per day for females and is equivalent to an increase of 3.9–5.2 g per day; and ‘target intake’ is an increase in current dietary intake to 38 g per day for males and 28 g per day for females and is equivalent to an increase of 6.9–13.2 g per day. All scenarios increase fibre intake using cereal fibre. ^†^ People in quartile 4 consumed in excess of the adequate and target levels of dietary fibre, and hence healthcare expenditure savings are not expected from this group. When analysed by quartiles of dietary fibre intake, the potential healthcare expenditure savings for CVD ranged from $127.9 million for 10% higher intake and up to $1.1 billion for the target intake. For T2D, the potential healthcare expenditure savings ranged from $35.1 million for 10% higher intake and up to $318.8 million for the target intake. The highest quartile of dietary fibre intake was not considered to contribute to any savings in healthcare expenditure given that both men and women in the highest quartile of intake consumed, on average, higher than the target intake.

**Table 3:** Productivity cost savings for cardiovascular disease and type 2 diabetes (AUD $m).

Scenario for Increased Intake of Fibre	Socio-Economic Status						Dietary Fibre Intake				
	Total	Quintile 1 (Lowest SES)	Quintile 2	Quintile 3	Quintile 4	Quintile 5 (Highest SES)	Total	Quartile 1 (Lowest)	Quartile 2	Quartile 3	Quartile 4 (Highest) ^†^
Cardiovascular Disease											
Persons											
10% higher	134.8	33.8	29.0	26.7	21.8	23.6	57.0	11.2	19.2	26.6	-
Adequate	278.8	89.1	67.1	52.8	39.3	30.6	347.4	195.4	119.7	32.4	-
Target	609.5	176.1	139.7	117.7	91.5	84.5	590.2	285.4	194.8	110.0	-
Males											
10% higher	75.9	19.2	16.3	15.0	12.4	13.0	32.8	6.4	11.0	15.4	-
Adequate	164.1	50.3	39.3	31.7	22.3	20.5	211.5	115.4	75.6	20.5	-
Target	410.3	115.1	93.3	80.0	61.2	60.7	398.2	184.6	132.1	81.2	-
Females											
10% higher	58.9	14.6	12.7	11.7	9.4	10.5	24.2	4.7	8.2	11.3	-
Adequate	114.7	38.8	27.8	21.1	17.0	10.0	135.9	80.0	44.1	11.9	-
Target	199.2	61.1	46.4	37.6	30.3	23.8	192.0	100.8	62.7	28.5	-
Type 2 Diabetes											
Persons											
10% higher	302.6	88.3	57.3	65.5	52.1	39.4	175.0	34.3	58.8	81.8	-
Adequate	652.6	236.3	135.5	132.5	95.4	52.9	1,009.6	576.5	331.5	101.6	-
Target	1,421.9	470.3	283.7	297.3	224.2	146.4	1,586.5	768.8	523.8	293.9	-
Males											
10% higher	182.9	53.8	34.6	39.4	31.7	23.4	105.0	20.7	35.2	49.2	-
Adequate	405.9	142.3	84.3	84.4	57.6	37.3	625.7	352.1	207.1	66.5	-
Target	1,001.8	323.5	199.2	212.0	157.5	109.7	1,072.6	501.0	356.0	215.5	-
Females											
10% higher	119.8	34.5	22.7	26.1	20.4	16.0	69.9	13.7	23.7	49.2	-
Adequate	246.7	94.0	51.2	48.1	37.7	15.7	383.9	224.5	124.4	35.0	-
Target	420.0	146.8	84.5	85.3	66.8	36.7	513.9	267.8	167.8	78.4	-

Abbreviations: SES, socioeconomic status. ‘10% higher’ is a 10% increase in current fibre intakes and is equivalent to an increase of between 2.1–2.5 g per day; ‘adequate intake’ is an increase in current dietary fibre intake to 30 g per day for males and 25 g per day for females and is equivalent to an increase of 3.9–5.2 g per day; and ‘target intake’ is an increase in current dietary intake to 38 g per day for males and 28 g per day for females and is equivalent to an increase of 6.9–13.2 g per day. All scenarios increase fibre intake using cereal fibre. ^†^ People in quartile 4 consumed in excess of the adequate and target levels of dietary fibre, and hence healthcare expenditure savings are not expected from this group.

**Table 4:** Total economic savings by population uptake for cardiovascular disease and type 2 diabetes (AUD $m).

	Cardiovascular Disease			Type 2 Diabetes		
Total economic savings ^†^	10% higher	Adequate	Target	10% higher	Adequate	Target
Healthcare productivity savings						
Universal	221.5	467.3	1026.5	60.7	131.0	285.9
Optimistic	110.8	233.7	513.3	30.4	65.5	143.0
Pessimistic	33.2	70.1	154.0	9.1	19.6	42.9
Very pessimistic	11.1	23.4	51.3	3.0	6.5	14.3
Productivity cost savings						
Universal	134.8	278.8	609.5	302.6	652.6	1,421.9
Optimistic	67.4	139.4	304.7	151.3	326.3	710.9
Pessimistic	20.2	41.8	91.4	45.4	97.9	213.3
Very pessimistic	6.7	13.9	30.5	15.1	32.6	71.1
Combined savings						
Universal	356.3	746.1	1636.0	363.4	783.6	1707.8
Optimistic	178.2	373.1	818.0	181.7	391.8	853.9
Pessimistic	53.4	111.9	245.4	54.5	117.5	256.2
Very pessimistic	17.8	37.3	81.8	18.2	39.2	85.4

‘10% higher’ is a 10% increase in current fibre intakes and is equivalent to an increase of between 2.1–2.5 g per day; ‘adequate intake’ is an increase in current dietary fibre intake to 30 g per day for males and 25 g per day for females and is equivalent to an increase of 3.9–5.2 g per day; and ‘target intake’ is an increase in current dietary intake to 38 g per day for males and 28 g per day for females and is equivalent to an increase of 6.9–13.2 g per day. All scenarios increase fibre intake using cereal fibre. ^†^ Universal, optimistic, pessimistic and very pessimistic scenarios assume 100%, 50%, 15% and 5% of adults increase their fibre intake using cereal fibre, respectively.

## Supplementary Materials

The following are available online at www.mdpi.com/2072-6643/10/1/34/s1, Table S1: Estimated number of cases averted for cardiovascular disease and type 2 diabetes, Table S2: Estimated cost per case for cardiovascular disease and type 2 diabetes (AUD$), 2015–16, Table S3: Categories of savings for cardiovascular disease and type 2 diabetes (AUD $m), Table S4: Savings for cardiovascular disease and type 2 diabetes per *g* of cereal fibre (AUD $m).
