# Supplementary material for: Healthcare Expenditure and Productivity Cost Savings from Reductions in Cardiovascular Disease and Type 2 Diabetes Associated with Increased Intake of Cereal Fibre among Australian Adults: A Cost of Illness Analysis
Source: Nutrients. 2018 Jan 2;10(1):34. doi: 10.3390/nu10010034 (PMC5793262; doi:10.3390/nu10010034)
Supplement: Supplementary file 1 [file nutrients-10-00034-s001.zip › Supplementary_Table_1-3.docx]

**Supplementary Table 1:** Estimated number of cases averted for cardiovascular disease and type 2 diabetes.

| **Cost category** | **10% higher** | **Adequate** | **Target** |
| --- | --- | --- | --- |
| **Cardiovascular Disease** | | | |
| Persons | 30,378  *(2.5%)* | 63,978  *(5.3%)* | 139,652  *(11.6%)* |
| Men | 17,649  *(1.5%)* | 38,576  *(3.2%)* | 95,927  *(7.9%)* |
| Women | 12,729  *(1.1%)* | 25,402  *(2.1%)* | 43,725  *(3.6%)* |
| **Type 2 Diabetes** | | | |
| Persons | 58,278  *(5.7%)* | 125,534  *(12.3%)* | 272,102  *(26.6%)* |
| Men | 34,354  *(3.4%)* | 76,251  *(7.5%)* | 188,197  *(18.4%)* |
| Women | 23,924  *(2.3%)* | 49,283  *(4.8%)* | 83,906  *(8.2%)* |

‘10% higher’ is a 10% increase in current fibre intakes and is equivalent to an increase of between 2.1-2.5 grams per day; ‘adequate intake’ is an increase in current dietary fibre intake to 30 grams per day for males and 25 grams per day for females and is equivalent to an increase of 3.9-5.2 grams per day; and ‘target intake’ is an increase in current dietary intake to 38 grams per day for males and 28 grams per day for females and is equivalent to an increase of 6.9-13.2 grams per day. All scenarios increase fibre intake using cereal fibre.

The number in brackets is the estimated reduction in disease prevalence.
