# Supplementary material for: Healthcare Expenditure and Productivity Cost Savings from Reductions in Cardiovascular Disease and Type 2 Diabetes Associated with Increased Intake of Cereal Fibre among Australian Adults: A Cost of Illness Analysis
Source: Nutrients. 2018 Jan 2;10(1):34. doi: 10.3390/nu10010034 (PMC5793262; doi:10.3390/nu10010034)
Supplement: Supplementary file 1 [file nutrients-10-00034-s001.zip › Supplementary_Table_2-3.docx]

**Supplementary Table 2:** Estimated cost per case for cardiovascular disease and type 2 diabetes (AUD$), 2015-16.

|  | **Cost per case ($)** | | |
| --- | --- | --- | --- |
| **Cost category** | **Persons** | **Males** | **Females** |
| **Cardiovascular Disease** | | | |
| **Total direct healthcare expenditure** | **7,286.96** | **7,555.95** | **6,971.23** |
| Hospital-admitted patient services | 4,273.48 | 4,810.75 | 3,642.87 |
| Out-of-hospital medical expenses | 1,434.39 | 1,286.53 | 1,607.94 |
| Prescription pharmaceuticals | 1,579.08 | 1,458.66 | 1,720.42 |
| **Total indirect productivity costs** | **45,399.20** | - | - |
| Reduced employment | 980.59 | - | - |
| Premature death | 42,274.30 | - | - |
| Absenteeism | 42.82 | - | - |
| Presenteeism | 2,101.49 | - | - |
| **Type 2 Diabetes** | | | |
| **Total direct healthcare expenditure** | **1,038.31** | **1,077.54** | **990.75** |
| Hospital-admitted patient services | 615.97 | 657.53 | 565.57 |
| Out-of-hospital medical expenses | 198.15 | 206.53 | 188.08 |
| Prescription pharmaceuticals | 224.19 | 213.55 | 237.10 |
| **Total indirect productivity costs** | **5,178.72** | - | - |
| Reduced employment | 1,316.05 | - | - |
| Premature death | 255.13 | - | - |
| Absenteeism | 295.63 | - | - |
| Presenteeism | 3,311.90 | - | - |
