# Supplementary material for: Healthcare Expenditure and Productivity Cost Savings from Reductions in Cardiovascular Disease and Type 2 Diabetes Associated with Increased Intake of Cereal Fibre among Australian Adults: A Cost of Illness Analysis
Source: Nutrients. 2018 Jan 2;10(1):34. doi: 10.3390/nu10010034 (PMC5793262; doi:10.3390/nu10010034)
Supplement: Supplementary file 1 [file nutrients-10-00034-s001.zip › Supplementary_Table_4-3.docx]

**Supplementary Table 4:** Savings for cardiovascular disease and type 2 diabetes per gram of cereal fibre (AUD $m).

| **Cost category** | **Universal** | **Optimistic** | **Pessimistic** | **Very pessimistic** |
| --- | --- | --- | --- | --- |
| **Cardiovascular Disease** | | | | |
| **Total direct healthcare expenditure** | **96.5** | **48.3** | **14.5** | **4.8** |
| Hospital-admitted patient services | 56.7 | 28.4 | 8.5 | 2.8 |
| Out-of-hospital medical expenses | 18.8 | 9.4 | 2.8 | 0.9 |
| Prescription pharmaceuticals | 21.0 | 10.5 | 3.1 | 1.0 |
| **Total indirect productivity cost savings** | **58.9** | **29.5** | **8.8** | **2.9** |
| Increased employment | 13.0 | 6.5 | 2.0 | 0.7 |
| Premature death averted | 17.4 | 8.7 | 2.6 | 0.9 |
| Absenteeism reduced | 0.6 | 0.3 | 0.08 | 0.03 |
| Presenteeism reduced | 27.9 | 14.0 | 4.2 | 1.4 |
| **Type 2 Diabetes** | | | | |
| **Total direct healthcare expenditure** | **26.5** | **13.3** | **4.0** | **1.3** |
| Hospital-admitted patient services | 15.8 | 7.8 | 2.4 | 0.8 |
| Out-of-hospital medical expenses | 5.1 | 2.5 | 0.8 | 0.3 |
| Prescription pharmaceuticals | 5.7 | 2.9 | 0.9 | 0.3 |
| **Total indirect productivity cost savings** | **132.3** | **66.1** | **19.8** | **6.6** |
| Increased employment | 33.3 | 16.7 | 5.0 | 1.7 |
| Premature death averted | 6.5 | 3.3 | 1.0 | 0.3 |
| Absenteeism reduced | 7.6 | 3.8 | 1.1 | 0.4 |
| Presenteeism reduced | 84.8 | 42.4 | 12.7 | 4.2 |

Universal, optimistic, pessimistic and very pessimistic scenarios assume 100%, 50%, 15% and 5% of adults increase their fibre intake using cereal fibre, respectively.
